
JOURNAL INFORMATION
==============================
NLM Title Abbreviation: Biospektrum (Heidelb)
Journal ID: Biospektrum (Heidelb)
NLM Journal ID: 9615685

Biospektrum

ISSN: 0947-0867
EISSN: 1868-6249

ARTICLE INFORMATION
==============================
PMCID: PMC8960677
PMID: 35369111
DOI: 10.1007/s12268-022-1729-2
Article ID: 1729
Article version: 1
Subjects: Wissenschaft · Special: Virologie

Nanoschalen aus DNA fangen und neutralisieren Viren

Sigl Christian
Dietz Hendrik dietz@tum.de

grid.6936.a 0000000123222966 Physik-Department, TU München, Am Coulombwall 4a, D-85748 Garching bei München, Deutschland
Electronic publication date: 2022 Mar 28
Print publication date: 2022
Volume: 28
Issue: 2
First page: 165
Last page: 167
Copyright: © Die Autoren 2022
License: Open Access: Dieser Artikel wird unter der Creative Commons Namensnennung 4.0 International Lizenz veröffentlicht, welche die Nutzung, Vervielfältigung, Bearbeitung, Verbreitung und Wiedergabe in jeglichem Medium und Format erlaubt, sofern Sie den/die ursprünglichen Autor(en) und die Quelle ordnungsgemäß nennen, einen Link zur Creative Commons Lizenz beifügen und angeben, ob Änderungen vorgenommen wurden. Die in diesem Artikel enthaltenen Bilder und sonstiges Drittmaterial unterliegen ebenfalls der genannten Creative Commons Lizenz, sofern sich aus der Abbildungslegende nichts anderes ergibt. Sofern das betreffende Material nicht unter der genannten Creative Commons Lizenz steht und die betreffende Handlung nicht nach gesetzlichen Vorschriften erlaubt ist, ist für die oben aufgeführten Weiterverwendungen des Materials die Einwilligung des jeweiligen Rechteinhabers einzuholen. Weitere Details zur Lizenz entnehmen Sie bitte der Lizenzinformation auf http://creativecommons.org/licenses/by/4.0/deed.de.
License URL: https://creativecommons.org/licenses/by/4.0/

issue-copyright-statement: © The Author(s), under exclusive licence to Springer-Verlag GmbH Deutschland, ein Teil von Springer Nature 2022

==============================
The current pandemic has highlighted the need for new antiviral therapies, to respond to existing and emerging diseases transmitted by viral vectors. We developed a novel antiviral approach that is based on neutralizing viruses by trapping and encapsulation in artificial nano-shells. The surrounding shells prevent the interaction of viruses with host cells and thus interrupt an essential step in the lifecycle of most viruses.

■ Die aktuelle SARS-CoV-2-Pandemie zeigt, welche Auswirkungen Viren auf unser tägliches Leben haben können. Weltweit haben sich mehr als 400 Millionen Menschen mit dem SARS-CoV-2-Virus infiziert, teilweise mit einem sehr schweren Krankheitsverlauf. Mehr als fünf Millionen Menschen sind bisher mit einer solchen Infektion gestorben [1]. Neben Coronaviren existieren viele weitere Viren, gegen die es bisher keine wirksame Behandlung gibt. Bereits vor der Covid-Pandemie starben jedes Jahr Millionen von Menschen an Virusinfektionen. Um auf virale Ausbrüche reagieren zu können, sind neue antivirale Therapien dringend notwendig.

Viren sind Krankheitserreger, die sich nicht selbst vermehren können und daher die Replikationsmaschinerie ihrer Wirtszellen ausnutzen. Ein wichtiger Schritt im Lebenszyklus von Viren ist die Bindung an Rezeptoren auf der Zellmembran (**Abb. 1**, links), gefolgt von dem Eindringen des Virus in die Wirtszelle, in der dann das Virus reproduziert wird. Wird ein Viruspartikel am Bindungsprozess gehindert, führt dies zur Unterbrechung des Lebenszyklus und damit zur Eindämmung der viralen Reproduktion.

Einige neutralisierende Antikörper unseres Immunsystems bekämpfen Virusinfektionen letztlich auf ähnliche Weise. Sie blockieren das Binden von Viren an Zellrezeptoren und verhindern dadurch die Aufnahme des Virus in die Zelle. Neutralisierende Antikörper müssen aber erst vom Immunsystem entwickelt und produziert werden. Dies dauert typischerweise einige Tage. Bei dem ersten Kontakt zu einem neuen Virus kommt es daher nicht sofort zu einer entsprechenden Immunantwort und das Virus kann sich schlimmstenfalls zunächst ungehindert im Körper ausbreiten. Zusätzlich wird die Wirksamkeit von bereits gebildeten Antikörpern, z. B. solche, die durch Impfungen gebildet werden, häufig durch Virusmutationen reduziert.

## Neuartige antivirale Plattform

Unser Konzept basiert auf der Neutralisation von Viren, indem sie in künstlichen Schalen aus DNA eingefangen und verkapselt werden. Diesen Ansatz kann man als eine Art Quarantänemaßnahme interpretieren, die auf der Ebene einzelner Viren wirkt. Viren, die von einer Nanoschale gefangen sind, werden analog der Funktionsweise vieler neutralisierender Antikörper an der Bindung an Rezeptoren auf der Zellmembran gehindert (**Abb. 1**, rechts).

Dieses antivirale Konzept bietet einige Vorteile gegenüber existierenden antiviralen Ansätzen. Viele antivirale Medikamente greifen in grundlegende Stoffwechselprozesse innerhalb der Zellen ein. Außerdem können sie oft nur in geringen Konzentrationen eingesetzt werden, um Nebenwirkungen zu reduzieren. Im Gegensatz dazu wirken die antiviralen Schalen außerhalb von Zellen und greifen nicht in zelluläre Prozesse ein. Ein weiterer Vorteil besteht darin, dass keine detaillierten Kenntnisse über die Struktur des Zielvirus erforderlich sind. Herkömmliche Inhibitoren müssen genau an infektionsrelevante Merkmale von Viren binden und sind dadurch im Falle von Virusmutationen besonders anfällig, ihre Wirkung zu verlieren. Bei den antiviralen Schalen spielen Details der Bindung keine Rolle, da das Schalenmaterial und nicht die Virusbinder das Virus neutralisieren. Es kann eine Vielzahl verschiedener virusbindender Moleküle an die Innenseite der Schalen montiert werden. Diese müssen dabei ihrerseits keine besonders hohe Affinität oder sogar neutralisierende Wirkung besitzen. Dieselbe Schalenplattform kann durch Wahl geeigneter virenbindender Moleküle gegen ein breites Spektrum verschiedener Viren eingesetzt werden.

## Konstruktion der Nanoschalen

Um unseren Ansatz zu realisieren, entwickelten wir ein neuartiges Nanofabrikationsverfahren, mit dem Schalen konstruiert werden können, die groß genug sind, um ganze Viren in ihrem Inneren zu fangen [2]. Das Designkonzept haben wir uns direkt aus der Natur abgeschaut: wir verwenden die gleichen Symmetrieprinzipien, die auch für ikosaedrische Viruskapside gelten und bereits

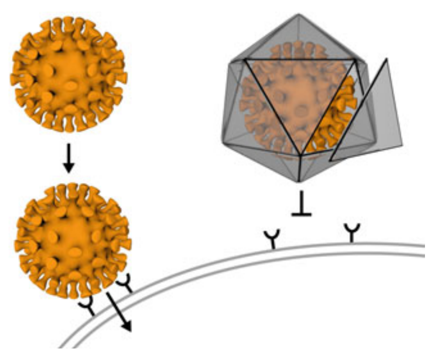

▲ **Abb. 1:** Schematische Darstellung der Bindung eines Virus an Zellrezeptoren (links) sowie der Neutralisation eines Virus durch eine Nanoschale, die das Binden an Zellrezeptoren verhindert (rechts).

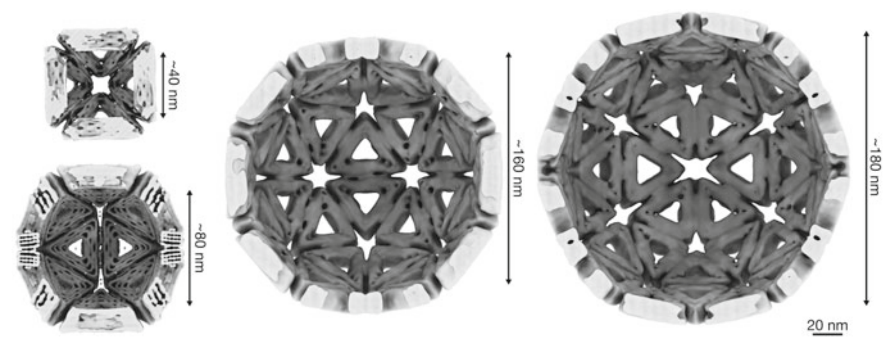

▲ **Abb. 2:** Schnitte durch die Struktur von DNA-Origami-Nanoschalen, rekonstruiert mit Kryo-Elektronenmikroskopie.

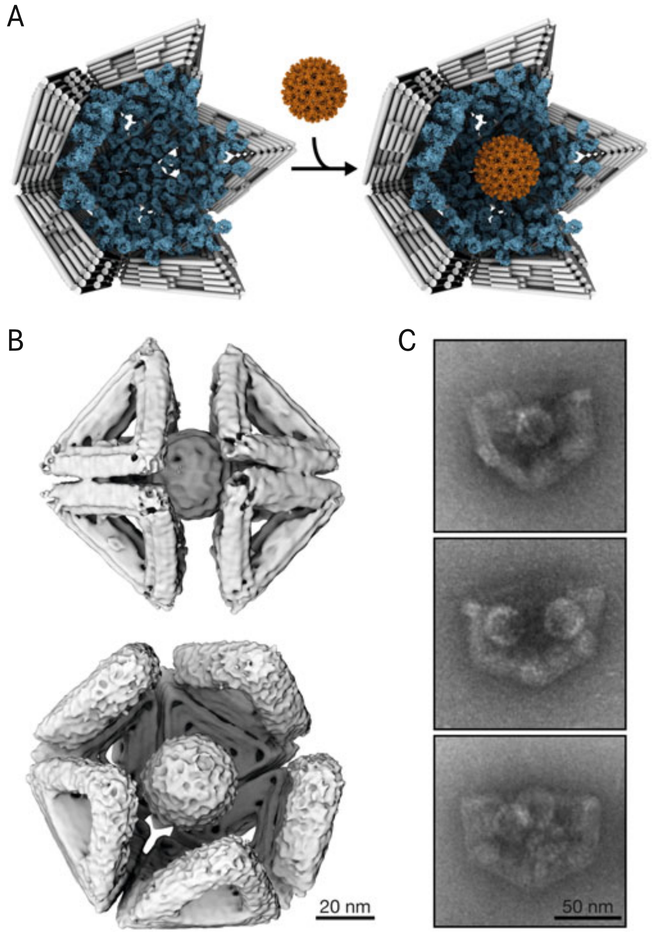

◀ **Abb. 3:** Nanoschalen schließen HBV-Kernpartikel ein. **A**, schematische Darstellung von Nanoschalen (weiß) mit bis zu 90 Antikörpern auf der Innenseite (blau), die ein HBV-Kernpartikel (orange) fangen. **B**, experimentelle Strukturen von verschiedenen Schalen, untersucht mit Kryo-Elektronenmikroskopie. **C**, Transmissions-Elektronenmikroskopie-Bilder von Nanoschalen, die bis zu drei HBV-Kernpartikel einschließen.

1962 von Caspar und Klug beschrieben wurden [3]. Die Schalen setzen sich aus mehreren dreieckigen Untereinheiten zusammen. Abhängig vom Design der Dreiecke können Schalen mit Durchmessern von 40 bis zu 180 Nanometern konstruiert werden (**Abb. 2**). Die Durchmesser der Schalen decken die Größe der meisten Viren ab. Zusätzlich können durch das rationale Design der Untereinheiten Schalen mit Öffnungen geformt werden, die das Fangen von Viren in den Schalen ermöglichen.

Die dreieckigen Untereinheiten werden mit der Methode des DNA-Origami geformt [4, 5]. Dabei wird ein langer einzelsträngiger DNA-Gerüststrang von mehreren kleineren Klammeroligonukleotiden in die gewünschte Form gebracht. Die Form der Struktur wird durch die DNA-Sequenzen der Klammeroligonukleotide bestimmt und kann je nach Anwendung benutzerdefiniert variiert werden. Mit dieser Methode können nanometergroße Strukturen mit fast beliebiger Funktion und Form geformt werden.

## Nanoschalen fangen und neutralisieren Viren

Um Viren in den Schalen einschließen und schließlich neutralisieren zu können, muss der Hohlraum der DNA-Schalen mit virusbindenden Molekülen ausgekleidet werden. Dabei können verschiedene funktionale Gruppen im Inneren der Schalen befestigt werden. Der Hohlraum des halben Ikosaeders besitzt bis zu 90 Befestigungsstellen (**Abb. 3A**). Dies hat aufgrund der Multivalenz eine sehr starke Bindung der Viren zur Folge. Diese starke Bindung bleibt auch bei der Verwendung von Virusbindern mit einer geringen Bindeaffinität bestehen.

Durch das Anbringen von Antikörpern gegen das Hepatitis-B-Virus(HBV)-Kernpartikel auf der Innenseite der Schalen konnten wir zeigen, dass HBV-Kernpartikel effizient in den Schalen eingeschlossen werden. Die experimentelle Struktur von eingeschlossenen Viren wurde mit Kryo-Elektronenmikroskopie untersucht (**Abb. 3B**). Das halbe Oktaeder ist zu klein, um das HBV-Kernpartikel vollständig zu bedecken. Deshalb binden zwei Halbschalen das Virus von zwei gegenüberliegenden Seiten. Bei größeren Schalen reicht eine Schale aus, um ein HBV-Partikel einzufangen. Es können sogar mehrere Viruspartikel in einer Schale gefangen werden (**Abb. 3C**).

Neben HBV-Partikeln konnten wir auch Adeno-assoziierte Viren (AAVs) in den Schalen fangen. In Zellkulturexperimenten konnten wir zeigen, dass eine Infektion von Zellen durch das Einschließen der AAV verhindert wird [2]. AVV und HBV gehören zu zwei verschiedenen Virusfamilien. Die Tatsache, dass zwei völlig verschiedene Virenpartikel gefangen werden können, lässt auf die generische Anwendung der Schalen schließen.

Der nächste Schritt bei der Entwicklung unserer antiviralen Plattform ist, die antiviralen Schalen in lebenden Organismen gründlich auf Verträglichkeit und Wirksamkeit zu testen. Im Erfolgsfall hat unser antivirales Konzept ein enormes Potenzial. Durch die Möglichkeit, die Schalen an neu auftretende Viren oder Virusmutationen anzupassen, steht mit unseren Virenfallen ein Wirkstoffansatz zur Verfügung, mit dem schnell auf zukünftige Virusausbrüche reagiert werden kann. Außerdem können nun Wirkstoffe für viele existierende virale Infektionskrankheiten hergestellt werden, für die es bisher keinerlei Therapiemöglichkeiten gibt.

## AUTOREN

Hier steht
eine Anzeige.
Springer


Funding note: Open Access funding enabled and organized by Projekt DEAL.

Christian Sigl 2011–2017 Physikstudium an der TU München. 2022 Promotion am Lehrstuhl für Biomolekulare Nanotechnologie, Physikdepartment, TU München unter Leitung von Prof. Dr. H. Dietz.

Hendrik Dietz 2004 Physikdiplom an der LMU München. 2007 Promotion am Physikdepartment der TU München. 2007–2009 Postdoc am Dana Farber Cancer Institute der Harvard Medical School, Boston, USA. Seit 2009 Professor für Biophysik und Leiter des Lehrstuhls für Biomolekulare Nanotechnologie am Physikdepartment der TU München.

Danksagung

Dieses Projekt wurde vom Forschungs- und Innovationsprogramm Horizon 2020 der Europäischen Union im Rahmen der Vereinbarung Nr. 899619 finanziert. Für die hier geäußerten Ansichten sind ausschließlich die Autoren verantwortlich. Die EU-Kommission übernimmt keine Verantwortung für die Verwendung der enthaltenen Informationen.


Literatur

[1] WHO (2022) WHO Coronavirus (COVID-19) Dashboard. https://covid19.who.int (Zugegriffen: 20.02.2022)
[2] Sigl C Willner EM Engelen W Programmable icosahedral shell system for virus trapping Nat Mater 2021 20 1281 1289 10.1038/s41563-021-01020-4 34127822 PMC7611604
[3] Caspar DL Klug A Physical principles in the construction of regular viruses Cold Spring Harb Symp Quant Biol 1962 27 1 24 10.1101/SQB.1962.027.001.005 14019094
[4] Rothemund PW Folding DNA to create nanoscale shapes and patterns Nature 2006 440 297 302 10.1038/nature04586 16541064
[5] Douglas SM Dietz H Liedl T Self-assembly of DNA into nanoscale three-dimensional shapes Nature 2009 459 414 418 10.1038/nature08016 19458720 PMC2688462
